# Aortic Valve Calcification Score in Patients with Arterial Hypertension Environmentally Exposed to Tobacco Smoke

**DOI:** 10.1007/s12012-021-09677-8

**Published:** 2021-07-26

**Authors:** Paweł Gać, Adrian Martuszewski, Patrycja Paluszkiewicz, Małgorzata Poręba, Grzegorz Mazur, Rafał Poręba

**Affiliations:** 1 Department of Hygiene, Wroclaw Medical University, Mikulicza-Radeckiego 7, 50-368 Wroclaw, Poland; 2 Centre of Diagnostic Imaging, 4Th Military Hospital, Weigla 5, 50-981 Wroclaw, Poland; 3 Department of Pathophysiology, Wroclaw Medical University, Marcinkowskiego 1, 50-368 Wroclaw, Poland; 4 Department of Internal Medicine, Occupational Diseases and Hypertension, Wroclaw Medical University, Borowska 213, 50-556 Wroclaw, Poland

**Keywords:** Environmental tobacco smoke, Aortic valve calcification, Computed tomography, SHSES scale

## Abstract

The objective of our study was to determine the relationship between exposure to environmental tobacco smoke (ETS) and the value of the aortic valve calcification score (AVCS) in people suffering from arterial hypertension (AH). 107 non-smokers with AH (mean age 67.16 ± 8.48 years) were qualified for the study. The degree of exposure to ETS was assessed using the Second-hand Smoke Exposure Scale (SHSES) questionnaire. Study group was divided depending on ETS exposure: A—no exposure, B—low, C—medium and D—high. AVCS was measured based on the aortic valve plane multiplanar reconstruction from the non-contrast phase of the cardiac computed tomography. The Agatston algorithm was used, in which calcifications were considered changes with a density exceeding 130 HU. The mean AVCS value in the study group of patients was 213.59 ± 304.86. The AVCS was significantly lower in subgroup A than in subgroups C and D. In subgroup A, the lack of aortic valve calcification (AVCS = 0) was observed significantly more frequently than in subgroups C and D. There was a positive correlation between the number of SHSES points and the AVCS value (*r* = 0.37, *p* < 0.05). Based on the ROC curve, the SHSES value was determined as the optimal cut-off point for the prediction of AVCS = 0, amounting to 3 points. The accuracy of SHSES < 3 as the predictor of AVCS = 0 was set at 62.18%. Hypertensive patients have an unfavourable relationship between the amount of exposure to ETS, determined on the SHSES scale, and the AVCS value.

## Introduction

There are over 1.3 billion people smoking tobacco and over 8 million of them die every year due to tobacco smoke [1, 2]. Environmental tobacco smoke (ETS) exposure which is known as passive smoking or second-hand smoking is still a global major health problem. ETS causes more over 1.2 million premature deaths per year and there is no safe level of tobacco exposure for non-smoking people [2]. A study published in 2011on children and adults from 192 countries has shown that 40% children were exposed to second-hand exposure. At least 603,000 people, including children, are affected ETS [3]. Zheng et al. [4] in their study in 2018 on 4,742 non-current smokers concluded that 65% of them were exposed to ETS. We can also distinguish third-hand smokers who are involuntary affected by residual tobacco smoke pollutants, e.g. smoker’s hairs or clothes. It is worth noting that there are such phrases as “passive smoking”, “environmental tobacco smoke exposure” and “second-hand smoke”. Second-hand smoke (SHS) is a more precise term and the other two refer to a more broad definition [5].

There is a positive correlation between ETS and hypertension [6–8]. A study conducted on over 9,000 children in which in utero exposure to tobacco smoke was also considered has shown that the exposed have greater odds to hypertension, especially increase of systolic blood pressure. Increased risk of hypertension due to ETS exposure was found in girls, especially in those whose father was smoking tobacco [9]. Elevation of arterial blood pressure significantly increased the risk of adverse cardiovascular events [10]. Hypertension is also a risk factor for heart failure, atrial fibrillation and sudden cardiac death (SDC) [11]. Children exposed to high levels of tobacco smoke have prevalence eustachian tube dysfunction [12]. Passive smokers have increased likelihood incidence of type 2 diabetes mellitus (T2DM) and pre-diabetes [13–15]. According to the WHO data [16, 17], heart disease was the leading cause of death between 2000 and 2019. Hypertension is a high cardiovascular risk. ETS besides increasing odds to arterial hypertension (AH) also increase risk for other cardiovascular diseases (CVD) to passive smokers with already diagnosed hypertension [18]. Passive smokers with hypertension especially with higher body mass index (BMI) have increased epicardial adipose tissue (EAT) thickness. EAT is a deposit localised between visceral pericardium and myocardium and increases the risk of cardiovascular diseases [19]. Passive tobacco smoke is also a significant risk factor for coronary artery calcification which was measured using non-gated computed tomography (CT) scans [20]. Chronic ETS lead to a vicious cycle of endothelial-mediated and leukocyte oxidant stress which results in vascular dysfunction [21]. Tobacco smoke contain high concentration of nicotine which activates sympathetic neurotransmission and induces oxidative stress in vascular endothelium. These events lead to endothelial disfunction and atherosclerosis lesions [22, 23].

ETS exposure can be assessed via laboratory measurements using serum or urine biomarkers [24]. It is directly possible to measure the level of nicotine or cotinine which is its metabolite. It is possible to measure ETS in hair. Cotinine can be measured in saliva and the results can be correlated positively with the urinary level of this metabolite. Hair colour can affect the uptake of cotinine [25, 26]. Nicotine in hair is not metabolised. Cotinine in urine also can be correlated with nicotine in the skin of non-smoking people. In serum nicotine has a low half-life time (2 h), but cotinine can be a good alternative as a metabolite as it exists for a longer time in serum [27]. ETS can be indirectly estimated by measuring the tobacco smoke components in the air (sidestream smoke) or by assessing the exposure using questionnaire such as The Second-hand Smoke Exposure Scale (SHSES). This scale firstly requires information about specific laboratory techniques and researchers. Using validated questionnaire is cheaper and a non-invasive method [28–30].

SHSES is an 11-point questionnaire which assess the non-smoking adults’ exposure to tobacco smoke in different places: home, car, public places and work. This scale was validated by Vardavas et al. and correlated with hair nicotine levels. Respondent is asked about exposure to tobacco smoke and its level: duration of exposition, exposure frequency or number of cigarettes smoked next to him. Direct ETS measurement methods seem to be more accurate, but the questionnaire used in this study is more readily available, cheaper and also repeatable [30].

The most prevalent form of aortic stenosis is aortic valve calcification (AVC). Before aortic stenosis there is a long period with AVC [31]. Aortic stenosis leads to hypertrophy of cardiomyocytes, reduction in coronary flow and left ventricular dysfunction which can be a reason for AH and heart failure. Smoking is a risk factor of AVC. Using multidetector CT (MDCT), there is a possibility of quantitative measurement of AVC by assessing adjacent axial slices of aortic valve. The slices are then analyzed to identify pixels with a density greater than 130 Hounsfield units (HU); 4 adjacent such pixels, taking into account their anatomical location, can be considered as aortic valve calcification [32, 33]. The Agatston score is widely used in the scientific research and is a good method to assess calcification using MDCT [34]. Multiplanar reconstruction (MPR) allows to analyse images obtained in CT in different planes. Aortic valve calcification score (AVCS) is based on measuring the area of AVC in accordance with Agatston and is expressed in Agatston units per cm^2^ (AU). AVC is a predictor of the risk of stenosis progression and of mortality. In symptomatic patients AVCS using MDCT may be used for assessing severity of stenosis [32, 35, 36]. Christensen et al. [37] analysed AVC in patients with history of smoking tobacco and they highlighted that AVC in these patients has an impact on mortality.

The aim of this study was to determine the relationship between exposure to ETS estimated based on the SHSES and the value of the AVCS in people suffering from AH.

## Materials and Methods

The study group consisted of 107 non-smoking patients with diagnosed and pharmacologically managed hypertension. For this study 54 males and 53 females with the mean age of 67.16 ± 8.48 years (age range 38–87 years) were qualified. To be included in the study, participants must be aged at least 18 years old, be hypertension diagnosed and pharmacologically treated for ≥ 5 years, have indication for coronary computed tomography angiography (CCTA) and have no history of smoking cigarettes. The exclusion criteria were as follows: patients with secondary hypertension, previously diagnosed ischaemic heart disease, previous stroke, T2DM, hypothyroidism or hyperthyroidism, chronic kidney disease (CKD) and patients with insufficient quality of the coronary CTA. Group size was determined using sample size calculator. The selection conditions were as follows: population size 38 million, fraction size 0.4, maximum error 10% and confidence level 95%. The required minimum size of study group was 92. Table 1 summarises the clinical characteristics of our study group of patients.

**Table 1:** Clinical characteristics of the studied group

Total patients^a^	107/100.0
Age (years)^b^	67.16 ± 8.48
*Gender*	
Men^a^	54/50.5
Women^a^	53/49.5
Height (m)^b^	1.68 ± 0.08
Body mass (kg)^b^	73.29 ± 11.88
BMI (kg/m^2^)^b^	25.99 ± 3.29
*Essential hypertension*^a^ Systolic blood pressure (mmHg)^b^ Diastolic blood pressure (mmHg)^b^	107/100.0 138.97 ± 15.37 85.70 ± 9.99
*Hypotensive drugs*	
ACE inhibitors^a^	63/58.9
β-blockers^a^	46/43.0
Diuretics^a^	29/27.1
Calcium channel blockers^a^	34/31.8
Angiotensin receptor blockers^a^	11/10.3
*Lipid profile*	
Total cholesterol (mg/dl)^b^	223.82 ± 50.35
Triglycerides (mg/dl)^b^	189.59 ± 167.59
Fasting glucose (mg/dl)^b^	110.56 ± 40.80

*ACE* angiotensin-converting enzyme, *BMI* body mass index

^a^Number/percentages

^b^Arithmetic mean ± standard deviation

From each patient included in this study we collected medical history questionnaire, basic anthropometrics assessment, arterial blood pressure (BP) measurements, serum lipid profile (total cholesterol, triglycerides), fasting glucose in blood and questionnaire assessment of ETS and CCTA.

Arterial BP measurements were obtained by the Korotkov method. Serum lipid profile and glucose concentration were measured using standard methods described in the instructions provided with the ordered reagent kits.

**Fig. 1:**
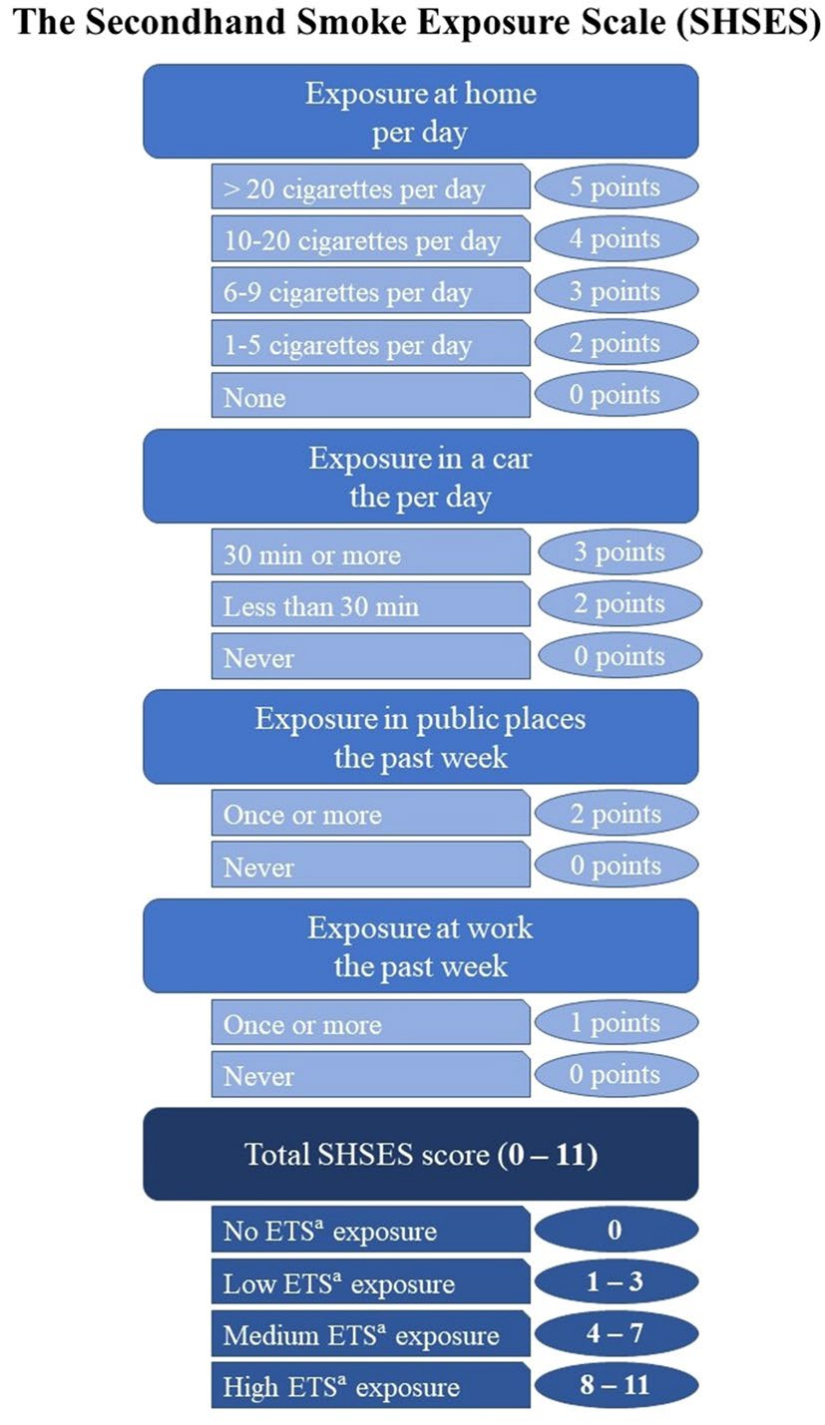
SHSES and its interpretation based on Vardavas et al. [29]; ^a^Environmental Tobacco Smoke (ETS)

The degree of exposure to ETS was estimated by SHSES which was used before by Vardavas et al. [30]. SHSES considers four different sites of exposure: home (per day), car (per day), public places (past week) and work (past week). Depending on the answer given, it is possible to obtain the appropriate number of points which is used in the assessment of ETS exposure. Using the before mentioned scale, the following subgroups of patients were distinguished: A—no ETS exposure (0 SHSES points, *n* = 51), B—low exposed to ETS (1–3 SHSES points, *n* = 14), C—medium exposed to ETS (4–7 SHSES points, *n* = 22) and D—high exposed to ETS (8–11 SHSES points, *n* = 20). Figure 1 presents SHSES and its interpretation.

Cardiac computed tomography (CCT) was also performed. Dual-source 128-slice CT scanner SOMATOM Definition Dual-Source CT (Siemens Healthcare, Germany) and standard protocol were used to perform the coronary computed tomography angiography (CCTA). Aortic valve calcification score (AVCS) was retrospectively measured using syngo.CT CaScoring (Siemens Healthcare, Germany). The AVCS was assessed semiautomatically, based on the multiplanar reconstruction (MPR) from the non-contrast phase of the CCT, made of 3.0-mm-thick layers in planes parallel to the aortic ring. The Agatston algorithm was used in which calcifications were considered changes with a density exceeding 130 HU. Lesions suggested by application as calcified were verified as lesions in the aortic valve leaflets/aortic ring (calcifications included in AVC) or lesions outside the previously mentioned structures (calcifications not included in AVC).

Ethical approval from the Local Ethics Committee was obtained. Written informed consent was obtained from all the study participants.

Statistical analysis was performed with the application "Dell Statistica" (Dell Inc., USA). Quantitative variables were presented as arithmetic means ± standard deviations. The distribution of variables was determined by the Shapiro–Wilk test. For normally distributed quantitative variables, the t test or ANOVA was used to test the hypotheses. For quantitative variables with no normal distribution, the Mann–Whitney U test or the Kruskal–Wallis ANOVA test was used to test the hypotheses. Qualitative variables were presented as counts/percentages. The Chi-square test was used in the comparative analysis of qualitative variables. To establish the relationship between the studied variables, a correlation analysis was performed. In the prediction analysis, the optimal predictor cut-off was determined based on the ROC curve. Predictor sensitivity and specificity analysis were also performed. The results at the level of p < 0.05 were considered statistically significant.

## Results

Related to SHSES 47.7% (*n* = 51) patients were not exposed to ETS and the remaining 52.3% (*n* = 56) were exposed. In public places 44.8% (*n* = 48) participants were exposed on ETS, at home 41.1% (*n* = 44) was exposed, in the car 34.6% (*n* = 37) and at work 26.2% (*n* = 28). Table 2 presents ETS exposure in the studied group based on SHSES.

**Table 2:** ETS exposure based on SHSES in the studied group

SHSES score^a^		3.13 ± 3.67
*ETS exposure based on SHSES*		
No^b^		51/47.7
Low^b^		14/13.1
Medium^b^		22/20.6
High^b^		20/18.7
*Exposure at home per day*		
> 20 cigarettes per day^b^		4/3.8
10–20 cigarettes per day^b^		16/14.9
6–9 cigarettes per day^b^		18/16.8
1–5 cigarettes per day^b^		6/5.6
None^b^		63/58.9
*Exposure in a car per day*		
30 min or more^b^		10/9.3
Less than 30 min^b^		27/25.2
Never^b^		70/65.4
*Exposure in public places the past week*		
Once or more^b^		48/44.8
Never^b^		59/55.1
*Exposure at work the past week*		
Once or more^b^		28/26.2
Never^b^		79/73.8

^a^Arithmetic mean ± standard deviation

^b^Number/percentages

The mean AVCS value in the study group of patients with AH was 213.59 ± 304.86. The maximal AVCS value was 1902.33 and the minimal 0.00. The median AVCS was 148.81 with interquartile range (IQR) 0.00–359.32. The AVCS = 0 involved 28.9% participants. The probability of severe aortic stenosis based on AVCS was mild amongst 98.1% participants (*n* = 105), moderate—0.9% (*n* = 1) and severe—0.9% (*n* = 1).

Participants with ETS exposure had higher AVCS (318.29 ± 351.89) than these without (125.37 ± 198.36) ETS exposure (*p* < 0.05). Statistically significant AVCS was increased in patients from subgroup C and D in relation to participants from subgroup A. In subgroup A, the lack of aortic valve calcification (AVCS = 0) was observed significantly more frequently than in subgroup C and D. An analysis was performed for the relationship between AVCS and the place of the exposure for each of locations mentioned in SHES. The mean AVCS value was the highest in patients exposed to ETS at work (408.35 ± 360.50) and the lowest in patients without exposure at home (141.10 ± 196.08). AVCS was statistically significantly higher in the subgroup of patients exposed to ETS at home than in patients not exposed to ETS at home; in the subgroup of patients exposed to ETS in a car than in patients not exposed to ETS in a car; in the subgroup of patients exposed to ETS in public places than in patients not exposed to ETS in public places; as well as in the subgroup of patients exposed to ETS at work than in patients not exposed to ETS at work. The analysis of AVCS in the subgroup based on ETS exposure is presented in Table 3.

**Table 3:** AVCS in subgroup divided based on ETS exposure characteristics

	AVCS ^a^	AVCS = 0 ^b^
*ETS exposure based on SHSES*		
No	125.37 ± 198.36*	27/52.9*
Yes	318.29 ± 351.89	4/7.1
*ETS exposure based on SHSES*		
No	125.37 ± 198.36**	27/52.9**
Low	206.59 ± 175.93	2/14.3
Medium	301.80 ± 430.16	1/4.6
High	452.70 ± 404.92	1/5.0
*Exposure at home*		
No	141.10 ± 196.08*	30/47.6*
Yes	348.39 ± 381.98	1/2.3
*Exposure in a car*		
No	147.14 ± 190.96*	29/41.4*
Yes	376.17 ± 407.74	2/5.4
*Exposure in public places*		
No	141.82 ± 199.40*	28/47.5*
Yes	330.23 ± 372.61	3/6.2
*Exposure at *work		
No	161.83 ± 253.33*	30/38.0*
Yes	408.35 ± 360.50	1/3.6

^a^ Arithmetic mean ± standard deviation

^b^ Number/percentages

* yes vs. no: *p* < 0.05; ** high vs. no and medium vs. no: *p* < 0.05

**Fig. 2:**
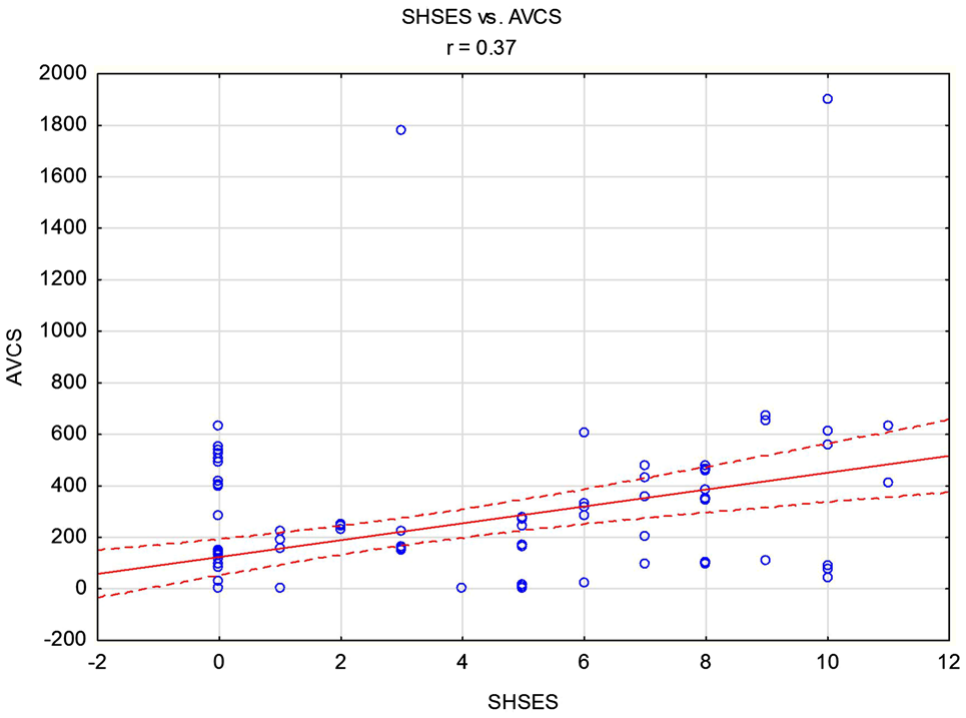
Correlation between SHSES score and AVCS

Moreover, there was a positive correlation between the number of SHSES points and the AVCS value (r = 0.37, *p* < 0.05), Fig. 2.

**Fig. 3:**
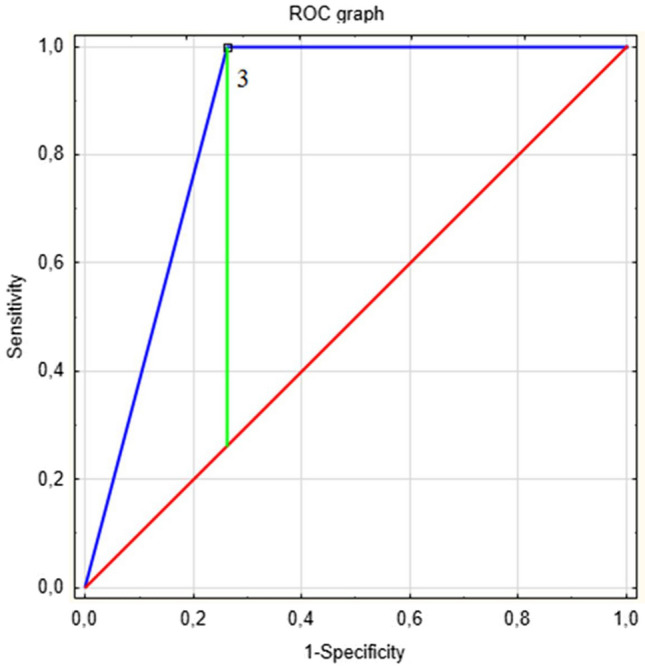
The ROC curve to predict AVCS = 0 based on the destimulant of the SHSES score

The ROC curve indicated a SHSES score value of 3 as an optimal cut-off point to predict AVCS = 0 in the studied group of patients. The criterion of SHSES score < 3 indicates AVCS = 0 with an accuracy of 62.2% (sensitivity 53.2% and specificity 100.0%). The discussed ROC curve to predict AVCS = 0 amongst patients is presented in Fig. 3, and the results of the performed accuracy analysis in Table 4.

**Table 4:** Sensitivity and specificity of the SHSES score < 3 points as a predictor AVCS = 0

Sensitivity	0.532
Specificity	1.000
Accuracy	0.622
Positive predictive values	0.938
Negative predictive values	0.475
Likelihood ratios positive	6.118
Likelihood ratios negative	0.452

## Discussion

Our study showed that in patients with AH there is a significant correlation between ETS exposure expressed using SHSES and AVC. To our knowledge this is the first study assessing ETS via questionnaire and AVC presence.

Calcification of aortic valve is not a passive process, but this is a result of multiple biological and molecular processes, inflammation and fibrosis. Hyperlipidaemia, especially elevated lipoprotein (a) level, also plays a role in calcification process by promoting local inflammation. Coronary artery atherosclerosis coexists in over 50% cases with AVC [38–40]. Firstly, there is a dysfunction of endothelium due to mechanical stress related to valves. Then there occur different changes in valvular endothelial and interstitial cells which result in apoptosis. This led to diffuse calcification which is stimulated by apoptosis of valvular interstitial cells. Mineral reabsorption is impaired due to insufficient function of cardiovascular osteoclast-like cells [41]. Aortic valve sclerosis can be auscultated in most cases of stenosis and visualised using echocardiography (ECHO) by finding focal areas of valve thickness and calcinosis. MDCT is a more precise diagnostic method and is useful whether ECHO is inconclusive or impossible [32]. Attard et al. [42] conducted a study in which they also analysed the correlation between ETS and risk of myocardial infarct or lipid profile. ETS, especially at home rather than at public places, was associated with a significantly increased risk of myocardial infarction. Active smokers and ETS had higher total cholesterol level. Passive smokers have also endothelial dysfunction in coronary artery [43]. In our study, we analysed the correlation between place of ETS exposure and AVCS. The mean value of AVCS was highest in patients exposed to ETS at work. Patients with exposure to ETS in public places had lower mean AVCS value than those exposed to ETS at home.

PROVIDI study group in their study [44] showed the prognostic value of incidental heart valve calcification detection on CVD. Utsunomiya et al. [45] analysed 943 patients to assess heart calcification and its association with CVD morbidity and mortality. Heart calcification, which also included AVC, increased the CVD morbidity and mortality compared to traditional risk factors. Patients with AVC have increased risk of coronary artery calcification (CAC) [46–48]. Mahabadi et al. [49] concluded that AVC is associated with increased coronary artery plaque (CAP). Their study group consisted of 357 participants and they used MDCT to assess the presence of CAP and AVC. Patients with AVC had increased extent of CAP (over 2 segments more) and higher frequency of CAP presence (89.2%, *p* < 0.001). These findings were not associated with sex, age and cardiovascular risk factors. Symptomatic AVC also predisposes to coronary events and worsens survival [48]. Individuals with CAC have high risk of coronary artery disease and CVD [50]. Aortic valve abnormality worsens patient’s prognosis. Aortic valve sclerosis may increase odds of stroke event [51]. Cardiovascular Heart Study [52], in which 5,201 people in age of at least 65 years were enrolled, showed that aortic valve disease morbidity was increased in case of AH and present smoking (20% and 35%, respectively, increase in risk). Progression of AVC is not significantly associated with stroke but with progression of mitral annular calcification (MAC) [53]. Other studies showed that there is no association between AVC and increased risk of stroke [54] or dementia [55]. Adler et al. [56] in their study confirmed that presence of AVC increases odds of death from CVD causes. There is a necessity of study to compare ETS and MAC. Our study showed that ETS is associated with the presence of AVC and thus increases, as literature shown, the risk of CVD.

Khurrami et al. [31] in their pilot study on 2,060 participants showed that left ventricular ejection fraction did not differ in case of low and high AVC. However, left ventricular and atrial hypertrophy were observed in patients with AVC regardless of the presence of the atrial stenosis symptoms. Nitta et al. [57] also concluded in their study that there is no correlation between the presence of AVC and left ventricular ejection fraction.

Lindroos et al. [58] showed that there is a correlation between AH and AVC and this does not depend on BMI and age. On the other hand, Owens et al. [59] concluded that BMI has an impact on incidence of AVC but not on its progression. Other retrospective study [60] showed that AH has an impact on rapid progression of AVC. Participants with hypertension had faster progression of atrial stenosis than these without AH (*p* < 0.01). AVC progression can be lower in patients with higher diastolic blood pressure [59]. Ljungberg et al. [61] claim that higher diastolic blood pressure in AH patients may be a risk factor of developing aortic stenosis requiring surgical interventions. These results are in line with study [62] on 3,474 individuals in which AH and smoking increased progression of AVC (*p* < 0.05). In our study patients with AH have higher AVCS. AVC development and progression correlate with smoking history especially with pack-years of smoking. The progression of AVC is increased with each 10-unit pack-year of smoking [63].

ETS is defined as the inhalation of smoke exhaled by the smoker and the smoke given off by the burning end of the tobacco product. The incidence of cardiovascular incidents in passive smokers is 20–50% higher than in unexposed individuals, significantly increasing mortality in the USA. There is scientific evidence for the association of SHS with carotid intima-media thickness, atherosclerotic plaque, arterial stiffness, endothelial function, and subclinical atherosclerosis. Frank Peinemann et al. [64] examined the effect of SHS on the degree of CAC. SHS was assessed by questionnaire, detailed history, laboratory and physical examination. CAC was determined by electron-beam CT and scored by Agatston and his co-workers. Total CAC score was calculated considering all calcified lesions in the coronary arteries. SHS had higher CAC values and CAC > 1 compared with unaffected subjects. SHS at home and work only led to increase in CAC + 1 of 17.9% and 52.1%, respectively. SHS may increase the risk of CVD. Yankelevitz et al. [20] used CAC assessment to investigate the presence of subclinical atherosclerosis in passive smokers who had never smoked and had no symptoms of coronary artery disease. There were 3,098 participants in the Flight Attendant Medical Research Institute International Early Lung Cancer Action Program. CAC was assessed by a low-dose non-gated CT. CAC was classified as absent, mild, moderate and severe calcification according to the number and length of epicardial arteries involved. The main, left anterior descending, circumflex and right coronary arteries were considered. CAC scores ranged from 0 to 12. There were also 4 categories of exposure to SHS: minimal, low, moderate and high exposure. The incidence of CAC was significantly higher in SHS with greater than minimal SHS exposure. The incidence and extent of CAC were dependent on SHS exposure. Similarly, in the other study performed by Yankelevitz et al. [65] the presence of coronary atherosclerosis in SHTS-exposed subjects was assessed using CT angiography. The study showed that the presence and extent of atherosclerosis depend on SHTS exposure.

A 2007 report from China's Ministry of Health [66] showed that 100,000 people die annually due to SHS. Based on the Guangzhou Biobank Cohort Study, active smoking was shown to be associated with aortic arch calcification (AAC), which is further associated with coronary artery disease. Most aortic calcifications are in the arch of aorta. Xu et al. [67] in their study wanted to assess whether ETS was a risk factor for AAC. The study group had plasma vascular risk factors measured. Calcification was assessed by radiographs taken by two radiologists. The length and width of calcifications were used to assess the factor of aortic arch calcification severity. In case of several calcification locations, the score was the sum of all calcification lengths. A 3-grade scale was created. Non-smoking patients were included in the study. When assessing ETS, the location and duration were considered. In women the risk of AAC increases with longer exposure to tobacco smoke. ETS is an independent risk factor for AAC. AAC is associated with a high incidence of coronary artery disease and is a highly specific marker of severe coronary atherosclerosis.

Knowledge regarding the cardiovascular consequences of smoking is limited. Aortic valve function in smokers may be adversely affected by mechanisms such as smoking-induced free radicals, altered lipoprotein composition, proinflammatory state and promotion of platelet activation. A study was conducted to evaluate the effect of smoking on degenerative aortic valve disease (DAVD) independent of other risk factors. Valve lesions were assessed by transthoracic echocardiography (TTE). DAVD was defined as abnormal irregular thickening of the valve leaflets with or without calcification. Twenty percent of subjects had DAVD, and slightly more than 40% with DAVD had calcification on a minimum of 2 aortic valve leaflets. The presence and severity of DAVD were found to depend on the dose and duration of smoking. The risk of DAVD decreased in subjects after smoking cessation and abstinence for at least 10 years. There is a need for further research on larger study groups to clarify the risk of progression over time of DAVD in relation to exposure to tobacco smoke [68]. Redd et al. [69] showed that both active and passive smoking are associated with cardiovascular risk factors. They also described an association between SHS and higher glucose levels, BMI, decreased HDL or decreased lung function as assessed by spirometry. There were differences in both sexes, especially in HDL levels. A stronger correlation between ETS and elevated blood glucose levels is found in men.

Recent scientific reports outline an important role for impaired autophagy in heart lesions caused by tobacco smoke exposure. A study was conducted to evaluate the effect of the mTOR-independent autophagy protein Beclin1 on cardiac changes caused by tobacco smoke exposure. The results showed that second-hand smoke induced excessive autophagy and activated the innate immune response. Beclin1 deficiency caused by tobacco smoke exposure led to changes in myocardial geometry, cardiomyocyte function and inflammation accompanied by marked apoptosis [70]. There are also scientific reports that macrophage migration inhibitory factor (MIF) may play a role in heart abnormalities caused by passive smoking. The effect of MIF knockout on changes in the heart caused by exposure to tobacco smoke has been investigated. The results showed that MIF knockout caused fractional shortening, impaired cardiomyocyte function, induced apoptosis and O_2_^−^ generation and excessive autophagolysosome formation [71]. Wang et al. [72] in a mouse study demonstrated that exposure to tobacco smoke alters the composition of the gut microbiota and reduces the inflammatory response. This was due to increased expression of tight junction proteins. Amongst the imaging studies used to evaluate aortic valve stenosis and calcification are transoesophageal echocardiography (TEE) and TTE. The diagnostic accuracy of both methods was compared. The reference point was intraoperative evaluation of the aortic valve. The study [73] included 169 patients without significant coronary artery disease. Systematic semi-quantitative assessment of AVC showed that real-time images by TTE and TEE were superior to visual or grey scale mean assessment of end-diastolic still frames. TTE is more accurate than TEE in assessing bicuspid aortic valve morphology (sensitivity and specificity 0.86, 92% and 94% versus 0.57, 77% and 82%, respectively). Multiplane transesophageal echocardiography is even more beneficial in assessing aortic valve morphology than the biplane approach. The sensitivity and specificity of the biplane technique were 66% and 56%, respectively, whereas in the multiplane technique (bicuspid vs tricuspid valve) it was 87% and 91%, respectively [74]. Zhu et al. [63] investigated the sensitivity and specificity of visual AVCs on low-dose CT (LDCT) by evaluating AVCs in lung cancer screening participants. The accuracy of the study using LDCT was assessed by comparing the results with standard-dose, electrocardiography (ECG)-gated CT. In moderate/severe AVC in moderate/severe AS, the sensitivity and specificity of LDCT were 100% and 94%, respectively. Our study showed 100% specificity for SHSES < 3 points.

Limitations of our study should be established. The first limitation is that this is a small study group and single-centre study. We have not got information about patients ETS based on laboratory test of nicotine or cotinine in saliva, plasma, urinary or hair as mentioned in other studies. SHSES scale was validated by Vardavas et al. (Pearson’s correlation: 0.4939, *p* < 0.0001) [30], but we do not know about honesty of patients in questionnaire filling. We did not check by validated scales the absence of dementia amongst them. The failure of measurement of biomarkers of exposure to tobacco smoke in biological material limits the reliability of the conclusions drawn in this study. The strength of our study is the use of CT instead of ECHO in detecting AVC. Moreover, we used MPR which is more precise than the single image and axial plane-based analysis. These modifications allowed better recognition of valve anatomical structures. In the present work, we did not differentiate the impact of other underlying factors including hypertension, high-cholesterol levels and interpersonal variability with ETS. The present results are aggregate of all factors; hence it is challenging to differential results that are specific to ETS. Further study could assess the association between AVC and ETS exposure measured by direct methods (e.g. cotinine in hair). Moreover, upcoming studies should analyse the correlation between SHSES and cotinine levels in hypertensive patients’ hair or saliva. The time since AH is diagnosed should also be included in further studies.

It can be concluded that in people with AH, there is an unfavourable relationship between the amount of exposure to environmental tobacco smoke, determined on the SHSES scale, and the intensity of the aortic valve calcification, expressed by the AVCS value.

## References

[1] Yousuf H, Hofstra M, Tijssen J, Leenen B, Lindemans JW, van Rossum A, Narula J, Hofstra L (2020). Estimated worldwide mortality attributed to secondhand tobacco smoke exposure, 1990–2016. JAMA Network Open.

[2] WHO. (n.d.). *Tobacco*. Retrieved May 4, 2021 from https://www.who.int/news-room/fact-sheets/detail/tobacco.

[3] Öberg M, Jaakkola MS, Woodward A, Peruga A, Prüss-Ustün A (2011). Worldwide burden of disease from exposure to second-hand smoke: A retrospective analysis of data from 192 countries. The Lancet.

[4] Zheng Y, Ji Y, Dong H, Chang C (2018). The prevalence of smoking, second-hand smoke exposure, and knowledge of the health hazards of smoking among internal migrants in 12 provinces in China: A cross-sectional analysis. BMC Public Health.

[5] Protano C, Vitali M (2011). The new danger of thirdhand smoke: Why passive smoking does not stop at secondhand smoke. Environmental Health Perspectives.

[6] Levy RV, Brathwaite KE, Sarathy H, Reidy K, Kaskel FJ, Melamed ML (2021). Analysis of active and passive tobacco exposures and blood pressure in US Children and Adolescents. JAMA Network Open.

[7] Akpa OM, Okekunle AP, Asowata JO, Adedokun B (2021). Passive smoking exposure and the risk of hypertension among non-smoking adults: The 2015–2016 NHANES data. Clinical Hypertension.

[8] Skipina TM, Soliman EZ, Upadhya B (2020). Association between secondhand smoke exposure and hypertension: Nearly as large as smoking. Journal of Hypertension.

[9] Zhang H, Yu L, Wang Q, Tao Y, Li J, Sun T, Zhang Y, Zhang H (2020). In utero and postnatal exposure to environmental tobacco smoke, blood pressure, and hypertension in children: The Seven Northeastern Cities study. International Journal of Environmental Health Research.

[10] Flint AC, Conell C, Ren X, Banki NM, Chan SL, Rao VA, Melles RB, Bhatt DL (2019). Effect of systolic and diastolic blood pressure on cardiovascular outcomes. New England Journal of Medicine.

[11] Kokubo Y, Matsumoto C, Islam MdS (2016). Hypertension is a risk factor for several types of heart disease: Review of prospective studies. Hypertension: From basic research to clinical practice.

[12] Patel MA, Mener DJ, Garcia-Esquinas E, Navas-Acien A, Agrawal Y, Lin SY (2016). Tobacco smoke exposure and eustachian tube disorders in US Children and Adolescents. PLoS ONE.

[13] Kowall B, Rathmann W, Strassburger K, Heier M, Holle R, Thorand B, Giani G, Peters A, Meisinger C (2010). Association of passive and active smoking with incident type 2 diabetes mellitus in the elderly population: The KORA S4/F4 cohort study. European Journal of Epidemiology.

[14] Bucheli JR, Manshad A, Ehrhart MD, Camacho J, Burge MR (2017). Association of passive and active smoking with pre-diabetes risk in a predominantly Hispanic population. Journal of Investigative Medicine.

[15] Sargeant LA, Khaw K-T, Bingham S, Day NE, Luben RN, Oakes S, Welch A, Wareham NJ (2001). Cigarette smoking and glycaemia: The EPIC-Norfolk Study. International Journal of Epidemiology.

[16] WHO. (n.d.). *WHO reveals leading causes of death and disability worldwide: 2000–2019*. Retrieved May 4, 2021 from https://www.who.int/news/item/09-12-2020-who-reveals-leading-causes-of-death-and-disability-worldwide-2000-2019.

[17] WHO. (n.d.). *Cardiovascular diseases (CVDs)*. Retrieved May 4, 2021 from https://www.who.int/news-room/fact-sheets/detail/cardiovascular-diseases-(cvds).

[18] Gać P, Jaźwiec P, Mazur G, Poręba R (2017). Exposure to cigarette smoke and the morphology of atherosclerotic plaques in the extracranial arteries assessed by computed tomography angiography in patients with essential hypertension. Cardiovascular Toxicology.

[19] Gać P, Czerwińska K, Poręba M, Macek P, Mazur G, Poręba R (2021). Environmental tobacco smoke exposure estimated using the SHSES scale and epicardial adipose tissue thickness in hypertensive patients. Cardiovascular Toxicology.

[20] Yankelevitz DF, Henschke CI, Yip R, Boffetta P, Shemesh J, Cham MD, Narula J, Hecht HS (2013). Second-hand tobacco smoke in never smokers is a significant risk factor for coronary artery calcification. JACC Cardiovascular Imaging.

[21] El-Mahdy MA, Abdelghany TM, Hemann C, Ewees MG, Mahgoup EM, Eid MS, Shalaan MT, Alzarie YA, Zweier JL (2020). Chronic cigarette smoke exposure triggers a vicious cycle of leukocyte and endothelial-mediated oxidant stress that results in vascular dysfunction. American Journal of Physiology-Heart and Circulatory Physiology.

[22] Argacha JF, Bourdrel T, van de Borne P (2018). Ecology of the cardiovascular system: A focus on air-related environmental factors. Trends in Cardiovascular Medicine.

[23] Leone A, Balbarini A (2008). Exposure to passive smoking: A test to predict endothelial dysfunction and atherosclerotic lesions. Angiology.

[24] Liu SH, Liu B, Sanders AP, Saland J, Wilson KM (2020). Secondhand smoke exposure and higher blood pressure in children and adolescents participating in NHANES. Preventive Medicine.

[25] Al-Delaimy WK (2002). Hair as a biomarker for exposure to tobacco smoke. Tobacco Control.

[26] Woodruff S, Conway T, Edwards C, Hovell M (2003). Acceptability and validity of hair collection from Latino children to assess exposure to environmental tobacco smoke. Nicotine & Tobacco Research.

[27] Torres S, Merino C, Paton B, Correig X, Ramírez N (2018). Biomarkers of Exposure to Secondhand and Thirdhand Tobacco Smoke: Recent Advances and Future Perspectives. International Journal of Environmental Research and Public Health.

[28] Swan GE, Lessov-Schlaggar CN (2007). The effects of tobacco smoke and nicotine on cognition and the brain. Neuropsychology Review.

[29] Collier AC, Pritsos CA (2003). Environmental tobacco smoke in the workplace: Markers of exposure, polymorphic enzymes and implications for disease state. Chemico-Biological Interactions.

[30] Vardavas C, Agaku I, Filippidis F, Kousoulis A, Girvalaki C, Symvoulakis E, Tzatzarakis M, Tsatsakis A, Behrakis P, Lionis C (2017). The Secondhand Smoke Exposure Scale (SHSES): A hair nicotine validated tool for assessing exposure to secondhand smoke among elderly adults in primary care. Tobacco Prevention & Cessation.

[31] Khurrami L, Møller JE, Dahl JS, Carter-Storch R, Christensen NL, Pareek M, Lindholt JS, Diederichsen ACP (2021). The association between aortic valve calcification, cardiovascular risk factors, and cardiac size and function in a general population. The International Journal of Cardiovascular Imaging.

[32] Lindman BR, Clavel M-A, Mathieu P, Iung B, Lancellotti P, Otto CM, Pibarot P (2016). Calcific aortic stenosis. Nature Reviews Disease Primers.

[33] Agatston AS, Janowitz WR, Hildner FJ, Zusmer NR, Viamonte M, Detrano R (1990). Quantification of coronary artery calcium using ultrafast computed tomography. Journal of the American College of Cardiology.

[34] Malguria N, Zimmerman S, Fishman EK (2018). Coronary artery calcium scoring: Current status and review of literature. Journal of Computer Assisted Tomography.

[35] *Rotterdam Coronary Artery Algorithm Evaluation Framework*. (n.d.). Retrieved May 4, 2021 from http://www.coronary.bigr.nl/stenoses/about.php.

[36] Clavel M-A, Messika-Zeitoun D, Pibarot P, Aggarwal SR, Malouf J, Araoz PA, Michelena HI, Cueff C, Larose E, Capoulade R, Vahanian A, Enriquez-Sarano M (2013). The complex nature of discordant severe calcified aortic valve disease grading. Journal of the American College of Cardiology.

[37] Christensen JL, Tan S, Chung HE, Ghosalkar DS, Qureshi R, Chu A, Yu W, Berus J, Shah NR, Wu W-C, Chun H, Aikawa E, Choudhary G, Morrison AR (2020). Aortic valve calcification predicts all-cause mortality independent of coronary calcification and severe stenosis. Atherosclerosis.

[38] Cho KI, Sakuma I, Sohn IS, Jo S-H, Koh KK (2018). Inflammatory and metabolic mechanisms underlying the calcific aortic valve disease. Atherosclerosis.

[39] Guddeti RR, Patil S, Ahmed A, Sharma A, Aboeata A, Lavie CJ, Alla VM (2020). Lipoprotein(a) and calcific aortic valve stenosis: A systematic review. Progress in Cardiovascular Diseases.

[40] Kamstrup PR, Tybjærg-Hansen A, Nordestgaard BG (2014). Elevated lipoprotein(a) and risk of aortic valve stenosis in the general population. Journal of the American College of Cardiology.

[41] Goody PR, Hosen MR, Christmann D, Niepmann ST, Zietzer A, Adam M, Bönner F, Zimmer S, Nickenig G, Jansen F (2020). Aortic valve stenosis: From basic mechanisms to novel therapeutic targets. Arteriosclerosis, Thrombosis, and Vascular Biology.

[42] Attard R, Dingli P, Doggen CJM, Cassar K, Farrugia R, Wettinger SB (2017). The impact of passive and active smoking on inflammation, lipid profile and the risk of myocardial infarction. Open Heart.

[43] Otsuka R (2001). Acute effects of passive smoking on the coronary circulation in healthy young adults. JAMA.

[44] Gondrie MJA, van der Graaf Y, Jacobs PC, Oen AL, Mali WPThM, On behalf of the PROVIDI Study Group (2011). The association of incidentally detected heart valve calcification with future cardiovascular events. European Radiology.

[45] Utsunomiya H, Yamamoto H, Urabe Y, Tsushima H, Kunita E, Kitagawa T, Hidaka T, Kihara Y (2013). Association between heart calcification assessed by echocardiography and future cardiovascular disease mortality and morbidity. IJC Heart & Vessels.

[46] Kälsch H, Lehmann N, Mahabadi AA, Bauer M, Kara K, Hüppe P, Moebus S, Möhlenkamp S, Dragano N, Schmermund A, Stang A, Jöckel K-H, Erbel R (2014). Beyond Framingham risk factors and coronary calcification: Does aortic valve calcification improve risk prediction? The Heinz Nixdorf Recall Study. Heart.

[47] Nasir K, Katz R, Al-Mallah M, Takasu J, Shavelle DM, Carr JJ, Kronmal R, Blumenthal RS, O’Brien K, Budoff MJ (2010). Relationship of aortic valve calcification with coronary artery calcium severity: The Multi-Ethnic Study of Atherosclerosis (MESA). Journal of Cardiovascular Computed Tomography.

[48] Kamperidis V, de Graaf MA, Broersen A, Ahmed W, Sianos G, Delgado V, Dijkstra J, Bax JJ, Scholte AJ (2014). Prognostic value of aortic and mitral valve calcium detected by contrast cardiac computed tomography angiography in patients with suspicion of coronary artery disease. The American Journal of Cardiology.

[49] Mahabadi AA, Bamberg F, Toepker M, Schlett CL, Rogers IS, Nagurney JT, Brady TJ, Hoffmann U, Truong QA (2009). Association of aortic valve calcification to the presence, extent, and composition of coronary artery plaque burden: From the Rule Out Myocardial Infarction using Computer Assisted Tomography (ROMICAT) trial. American Heart Journal.

[50] Hoffmann U, Massaro JM, D’Agostino RB, Kathiresan S, Fox CS, O’Donnell CJ (2016). Cardiovascular event prediction and risk reclassification by coronary, aortic, and valvular calcification in the Framingham Heart Study. Journal of the American Heart Association.

[51] Otto CM, Lind BK, Kitzman DW, Gersh BJ, Siscovick DS (1999). Association of aortic-valve sclerosis with cardiovascular mortality and morbidity in the elderly. New England Journal of Medicine.

[52] Stewart BF, Siscovick D, Lind BK, Gardin JM, Gottdiener JS, Smith VE, Kitzman DW, Otto CM (1997). Clinical factors associated with calcific aortic valve disease. This study was supported in part by Contracts NO1-HC85079 through HC-850086 from the National Heart, Lung, and Blood Institute, National Institutes of Health, Bethesda, Maryland. Journal of the American College of Cardiology.

[53] Fashanu OE, Bizanti A, Al-Abdouh A, Zhao D, Budoff MJ, Thomas IC, Longstreth WT, Michos ED (2020). Progression of valvular calcification and risk of incident stroke: The Multi-Ethnic Study of Atherosclerosis (MESA). Atherosclerosis.

[54] Bos D, Bozorgpourniazi A, Mutlu U, Kavousi M, Vernooij MW, Moelker A, Franco OH, Koudstaal PJ, Ikram MA, van der Lugt A (2016). Aortic valve calcification and risk of stroke: The Rotterdam study. Stroke.

[55] Wolters FJ, Bos D, Vernooij MW, Franco OH, Hofman A, Koudstaal PJ, van der Lugt A, Ikram MA, The Heart-Brain Connection Collaborative Research Group (2016). Aortic valve calcification and the risk of dementia: A population-based study. Journal of Alzheimer’s Disease.

[56] Adler Y, Shemesh J, Tenenbaum A, Hovav B, Fisman EZ, Motro M (2002). Aortic valve calcium on spiral computed tomography (dual slice mode) is associated with advanced coronary calcium in hypertensive patients. Coronary Artery Disease.

[57] Nitta K, Kurisu S, Erasta R, Ikenaga H, Ishibashi K, Fukuda Y, Kihara Y (2020). Aortic valve calcium is associated with left ventricular diastolic function in patients without evidence of ischaemic heart disease: Assessment by gated single-photon emission computed tomography. Acta Cardiologica.

[58] LlNDROOS M, Kupari M, Valvanne J, Strandberg T, Heikkilä J, TlLVIS R (1994). Factors associated with calcific aortic valve degeneration in the elderly. European Heart Journal.

[59] Owens DS, Katz R, Takasu J, Kronmal R, Budoff MJ, O’Brien KD (2010). Incidence and progression of aortic valve calcium in the multi-ethnic study of atherosclerosis (MESA). The American Journal of Cardiology.

[60] Capoulade R, Clavel M-A, Mathieu P, Côté N, Dumesnil JG, Arsenault M, Bédard É, Pibarot P (2013). Impact of hypertension and renin-angiotensin system inhibitors in aortic stenosis. European Journal of Clinical Investigation.

[61] Ljungberg J, Johansson B, Engström KG, Norberg M, Bergdahl IA, Söderberg S (2019). Arterial hypertension and diastolic blood pressure associate with aortic stenosis. Scandinavian Cardiovascular Journal.

[62] Abdalamir M, Goyfman M, Johnson D, Liu Y, Dabbous F, Chaus A, Budoff M (2019). The relationship between endothelial function and aortic valve calcification: multi-ethnic study of atherosclerosis. Atherosclerosis.

[63] Zhu Y, Wang Y, Gioia WE, Yip R, Jirapatnakul AC, Chung MS, Yankelevitz DF, Henschke CI (2020). Visual scoring of aortic valve calcifications on low-dose CT in lung cancer screening. European Radiology.

[64] Peinemann F, Moebus S, Dragano N, Möhlenkamp S, Lehmann N, Zeeb H, Erbel R, Jöckel K-H, Hoffmann B (2011). Secondhand smoke exposure and coronary artery calcification among nonsmoking participants of a population-based cohort. Environmental Health Perspectives.

[65] Yankelevitz DF, Cham MD, Hecht H, Yip R, Shemesh J, Narula J, Henschke CI (2017). The association of secondhand tobacco smoke and CT angiography-verified coronary atherosclerosis. JACC: Cardiovascular Imaging.

[66] *2007 China Tobacco Control Report*. (n.d.). Retrieved May 5, 2021 from https://www.tobaccofreekids.org/assets/global/pdfs/reports_articles/2007%20China%20MOH%20Tobacco%20Control%20Report.pdf.

[67] Xu L, Jiang CQ, Lam TH, Thomas GN, Zhang WS, Cheng KK (2011). Passive smoking and aortic arch calcification in older Chinese never smokers: The Guangzhou Biobank Cohort Study. International Journal of Cardiology.

[68] Dudzinski DM, O’Gara PT (2019). Association of cigarette smoking with degenerative aortic valve disease: Mediator or marker of risk?. Circulation: Cardiovascular Imaging.

[69] Reed RM, Dransfield MT, Eberlein M, Miller M, Netzer G, Pavlovich M, Pollin TI, Scharf SM, Shuldiner AR, Sin D, Mitchell BD (2017). Gender differences in first and secondhand smoke exposure, spirometric lung function and cardiometabolic health in the old order Amish: A novel population without female smoking. PLoS ONE.

[70] Liu F, Liu Y, Zhuang Z, Ma J, Xu X, Zhang W, Peng H, Yang L, Zhang W, Pei Z, Ren J (2020). Beclin1 Haploinsufficiency accentuates second-hand smoke exposure-induced myocardial Remodeling and contractile dysfunction through a STING-mediated mechanism. Journal of Molecular and Cellular Cardiology.

[71] Wang S, Chen X, Zeng B, Xu X, Chen H, Zhao P, Hilaire ML, Bucala R, Zheng Q, Ren J (2020). Knockout of macrophage migration inhibitory factor accentuates side-stream smoke exposure-induced myocardial contractile dysfunction through dysregulated mitophagy. Pharmacological Research.

[72] Wang H (2012). Side-stream smoking reduces intestinal inflammation and increases expression of tight junction proteins. World Journal of Gastroenterology.

[73] Yousry M, Rickenlund A, Petrini J, Jenner J, Liska J, Eriksson P, Franco-Cereceda A, Eriksson MJ, Caidahl K (2015). Aortic valve type and calcification as assessed by transthoracic and transoesophageal echocardiography. Clinical Physiology and Functional Imaging.

[74] Espinal M, Fuisz AR, Nanda NC, Aaluri SR, Mukhtar O, Sekar PC (2000). Sensitivity and specificity of transesophageal echocardiography for determination of aortic valve morphology. American Heart Journal.

